# Self-reported test ordering practices among Canadian internal medicine physicians and trainees: a multicenter cross-sectional survey

**DOI:** 10.1186/s12913-019-4639-3

**Published:** 2019-11-08

**Authors:** Thomas Bodley, Janice L. Kwan, John Matelski, Patrick J. Darragh, Peter Cram

**Affiliations:** 1 Department of Medicine, University of Toronto, Toronto, ON Canada; 2 Division of General Internal Medicine, Sinai Health System and University Health Network, Toronto, ON Canada; 3 Biostatistics Research Unit, University Health Network, Toronto, ON Canada; 4 Department of Medicine, Michael Garron Hospital, Toronto, ON Canada

**Keywords:** Diagnostic investigation, Quality improvement, Behavioural science, Hospital medicine

## Abstract

**Background:**

Over-testing is a recognized problem, but clinicians usually lack information about their personal test ordering volumes. In the absence of data, clinicians rely on self-perception to inform their test ordering practices. In this study we explore clinician self-perception of diagnostic test ordering intensity.

**Methods:**

We conducted a cross-sectional survey of inpatient General Internal Medicine (GIM) attending physicians and trainees at three Canadian teaching hospitals. We collected information about: self-reported test ordering intensity, perception of colleagues test ordering intensity, and importance of clinical utility, patient comfort, and cost when ordering tests. We compared responses of clinicians who self-identified as high vs low utilizers of diagnostic tests, and attending physicians vs trainees.

**Results:**

Only 15% of inpatient GIM clinicians self-identified as high utilizers of diagnostic tests, while 73% felt that GIM clinicians in aggregate (“others”) order too many tests. Survey respondents identified clinical utility as important when choosing to order tests (selected by 94%), followed by patient comfort (48%) and cost (23%). Self-identified low/average utilizers of diagnostic tests were more likely to report considering cost compared to high utilizers (27% vs 5%, *P* = 0.04). Attending physicians were more likely to consider patient comfort (70% vs 41%, *p* = 0.01) and cost (42% vs 17%, *p* = 0.003) than trainees.

**Conclusions:**

In the absence of data, providers seem to recognize that over investigation is a problem, but few self-identify as being high test utilizers. Moreover, a significant percentage of respondents did not consider cost or patient discomfort when ordering tests. Our findings highlight challenges in reducing over-testing in the current era.

## Background

Diagnostic investigations are instrumental in screening patients for disease, making a diagnosis, and monitoring response to therapy. In Ontario, Canada (population 13.5 million), hospital based laboratories process over 100 million laboratory tests annually (1), and from 2004 to 2012 the annual volume of computed tomography (CT) scans nearly doubled to over 1.5 million (2). Excessive testing is costly, (3) potentially harmful to patients and creates excess work for providers who must review and follow-up on ordered tests (4, 5). Excessive phlebotomy of hospitalised patients causes patient discomfort and iatrogenic anemia (6), while excess radiation exposure is known to increase cancer risk (7). Unnecessary testing can also lead to diagnostic error through incidental findings and “false positives” (8, 9), which can unleash a diagnostic cascade of further testing and unwarranted treatment (10). The combination of expense and patient harm has led groups such as Choosing Wisely to advocate for physician restraint in diagnostic testing of hospitalised patients (11).

Interestingly, while hospital based physicians are increasingly provided with individualized reports on readmission rates, hospital length-of-stay, and mortality, it is still uncommon for physicians to routinely receive data on their personal diagnostic test ordering practices (12, 13). Without data physicians must to rely on self-perception, though self-perceptions are known to be inaccurate in many settings (14, 15). We surveyed inpatient General Internal Medicine (GIM) attending physicians and trainees at three Canadian teaching hospitals to investigate self-perceived diagnostic test ordering intensity. We explore how a clinicians self-perception of their test ordering practices is influenced by their level of training, and how self-perception as a high or low utilizer of diagnostic tests is associated with different factors that physicians consider important when ordering tests.

## Methods

### Setting and participants

We conducted a survey of trainees (medical students and residents) and staff physicians (aka attendings) from inpatient GIM teaching services at three University of Toronto hospitals between November 2016 and October 2017. Study sites included Toronto General Hospital, Toronto Western Hospital, and Mount Sinai Hospital which are all tertiary/quaternary care hospitals in Toronto, Ontario. Attendings complete nearly all of their clinical work at their primary hospital, while trainees rotate between hospitals.

### Survey tool

We developed a survey tool (Additional file 1: Table S1) to investigate physician self-perceived diagnostic test ordering intensity, self-estimated test ordering volumes, and factors considered when ordering tests. Survey questions were developed via consensus by the study authors and refined using an iterative process. The survey was pilot tested with three colleagues and refined for usability, clarity, and content prior to distribution.

We collected respondent demographics including age, sex, and level-of-training/clinical experience. Using a five-point Likert scale with 1 representing negative responses (much lower, too few tests); 5 representing positive responses (much higher, too many tests), we asked respondents to: 1) rate their personal test ordering intensity relative to their GIM peers; 2) rate the test ordering intensity of their peers in aggregate; and 3) rate how often they considered patient comfort, cost, and clinical utility when deciding what tests to order. We also asked respondents to estimate the number of lab tests and imaging investigations (xrays, ECGs, MRIs, etc.) they order on a typical patient during the first 24 h of hospital admission and on follow-up over a 7 day hospitalization. Structured definitions of what we considered an investigation were provided (Additional file 1: Table S1).

### Sampling method and sample size

We invited all GIM attending physicians at the three study sites to participate through email. We surveyed a convenience sample of trainees; specifically, we distributed our survey to trainees on their GIM rotations at our participating hospitals who attended a series of eight noon teaching conferences between November 2016 and August 2017 that members of our study team attended. The survey was distributed to trainees attending the noon conferences and survey administration was followed by a 45-min teaching session led by members of our team for trainees on principles of diagnostic test stewardship, test result follow-up, and challenges. All surveys were completed anonymously using an implied consent process and without an incentive. We calculated that a sample size of 125 completed surveys would provide us with 80% power to detect a 0.5 difference in Likert responses for attendings compared to trainees. All data was stored, analyzed, and presented in aggregate. Institutional review board approval was obtained at each hospital site and the University of Toronto.

### Statistical analysis

Trainee response rate was calculated as the number of completed surveys divided by the total number of surveys distributed during noon conferences. Attending response rate was the number completed surveys divided by the number of attendings solicited through email. We used descriptive statistics to characterize respondent demographics. We compared responses of attendings versus trainees and self-identified low/average test utilizers (Likert 1–3) versus high test utilizers (4, 5) with respect to estimated test ordering volumes and the importance of patient comfort, test cost, and clinical utility using chi-square and Fisher’s exact tests for categorical variables, and t-tests for continuous variables. Chi-square statistics were used where samples sizes permitted, (16) with Fisher’s exact tests used for smaller cell sizes.

Recognizing that survey respondents may not complete all survey questions, we examined the proportion of missing data for each question (Additional file 2: Table S2). Percentages in the results are reported based on question specific response rates received for each survey item. We conducted subgroup analyses to evaluate potential differences in test ordering according to sex, level of training, attending experience (< 5 years vs > 5-years in practice), and after dichotomizing Likert responses into negative (Likert 1–2) and positive (Likert 4–5) responses, thereby removing intermediate responses (Likert 3). *P*-values are reported for all comparisons, and *p* < 0.05 were judged statistically significant. Statistical analyses were performed using Microsoft Excel 2013 (Microsoft Corp, Redmond, WA) and R Version 3.4.0 (R Core Team, Vienna, Austria).

## Results

The overall response rate was 83% (132/159); 92% (99/108) for trainees and 65% (33/51) for attendings (*p* < 0.001). Question specific response rates across all groups of respondents varied from 90 to 100% (Additional file 2: Table S2). The median age of attendings was 42 years (range 28 to 66 years, 38% female) and trainees was 27 years (range 23 to 37 years, 43% female).

**Fig. 1:**
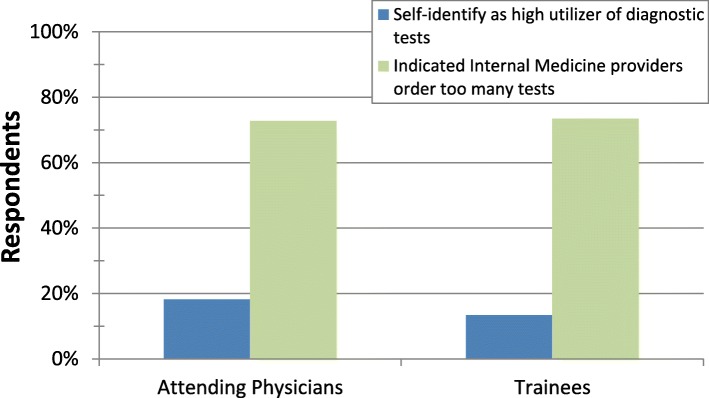
Percentage of attendings and trainees who self-identify as high utilizers of diagnostic tests (blue) and who identify high utilization as a problem among their peers (green)

Only 15% (19/130) of respondents self-identified as high utilizers of diagnostic tests relative to their peers, but 73% (96/131) felt that GIM physicians as a group ordered too many tests. The magnitude of this discrepancy was consistent between attendings and trainees (Fig. 1).

Respondents who self-identified as high utilizers of tests did not differ from low/average utilizers in their self-reported testing volumes, nor did estimated test ordering volumes differ between attendings and trainees (Table 1).

**Table 1:** Test ordering by self-identified high vs low/average utilizers of diagnostic tests and attending physicians vs trainees

	High Utilizers^*^ of Tests (*N* = 19)^**^	Low/Avg^*^ Utilizers of Tests (*N* = 111)^**^	p	Attending Physicians (*N* = 33)^**^	Trainees (*N* = 99)^**^	p
Median Age, number (min-max)	29 (25–48)	28 (23–66)	0.88	42 (28–66)	27 (23–37)	< 0.001
Female Sex, number (%)	11 (58%)	44 (40%)	0.15	12 (38%)	43 (43%)	0.55
Self-identified as a high utilizer of tests^†^, number (%)	–	–	–	6 (18%)	13 (13%)	0.57
Indicate that GIM providers order too many tests^†^, number (%)	14 (74%)	81 (73%)	0.95	24 (73%)	72 (73%)	0.93
Average number of lab tests per patient ordered in first 24 h of admission, number (SD)	12.4	9.9	0.12	12.5	9.5	0.10
Average number of other^‡^ tests ordered in first 24 h, number (SD)	2.8	2.7	0.88	2.9	2.7	0.26
Average number of daily lab tests per patient in first week of admission, number (SD)	4.4	4.6	0.88	4.8	4.5	0.66
Average estimated number of other^‡^ tests per day of admission, number (SD)	1.0	0.7	0.31	0.7	0.7	0.78
Feels confident when estimating number of lab and other tests ^†^, number (%)	2 (11%)	10 (9%)	0.69	3 (9%)	9 (9%)	1.00
Strongly considers cost when choosing lab tests^†^, number (%)	1 (5%)	30 (27%)	0.04	14 (42%)	17 (17%)	< 0.001
Strongly considers patient comfort when choosing lab tests^†^, number (%)	9 (47%)	54 (49%)	0.92	23 (70%)	41 (41%)	0.01
Strongly considers clinical utility when choosing lab tests^†^, number (%)	16 (84%)	104 (95%)	0.31	33 (100%)	89 (92%)	0.20
Proportion of work day spent deciding what tests to order, %	32%	27%	0.23	19%	31%	< 0.001

^*^High Utilizers of tests correspond to a 4 or 5 on 5-point Likert Scale when asked to rate their diagnostic test ordering intensity relative to their peers. Low/Average Utilizers correspond to a 1–3 on the same scale

^**^Percentages are based on question specific response rates rather than overall survey response rates

^†^Response corresponds to 4 or 5 on a 5-point Likert Scale

^‡^Structured definition for “other investigations” was provided including radiographic imaging, ECGs, etc.

SD = Standard Deviation

Among all respondents, 94% (122/130) considered clinical utility (i.e., whether a test would impact patient management) most of the time or always (4 or 5 on Likert scale) when deciding what tests to order. 48% (64/132) considered patient comfort most of the time or always and 23% (31/132) considered cost most of the time or always. Attendings were more likely than trainees to identify patient comfort (70% vs 41%, *p* = 0.01) and cost (42% vs 17%, *p* = 0.003) as important. Self-identified low/average test utilizers were more likely to identify cost as important compared to high utilizers (27% vs 5%, *P* = 0.04). Subgroup analyses yielded similar results and are available in Additional file 3: Table S3.

## Discussion

In a multi-center study of Canadian inpatient GIM physicians and trainees we investigated self-reported diagnostic test ordering practices. A majority of respondents (73%) identified their colleagues as ordering too many tests, but only 15% self-identified as high test utilizers themselves. We also found infrequent consideration of cost (23%) and patient comfort (48%) when deciding what tests to order. Despite significant efforts to increase awareness of diagnostic minimalism and resource stewardship (11), our findings suggest that important barriers to improvement remain.

Our finding that GIM providers identify their colleagues as high utilizers of diagnostic tests far more often than themselves is mathematically implausible, and may reflect the lack of real-time test ordering feedback to clinicians. GIM physicians in our hospitals receive group-level data on hospital length-of-stay, mortality, and readmission rate, but granular data on diagnostic testing intensity is not routinely available. A number of research teams have demonstrated that audit-and-feedback or computerized “dashboards” providing individualized diagnostic testing data can be helpful (13, 17). Diagnostic test management toolboxes propose interventions to help organizations improve test utilization (18), including individual physician test utilization report cards. While these practices hold promise, they are not widely implemented and a recognized limitation is the need to provide incentives to review and improve performance (18). Lack of real-time diagnostic testing intensity is likely to contribute to well recognized over-use of tests ranging from echocardiography to hemologbin A1c testing (19, 20).

Our findings are also consistent with concepts from behavioural psychology, where the “above average effect” or “comparative optimism effect” describes how individuals look favorably upon personal performance relative to peers (21). Comparative optimism has been shown in surgical residents who over-estimated their global performance (22), and may contribute to diagnostic error though physician anchoring/commitment to a misdiagnosis (23). Our study suggests that self-perceptions are likely to be inaccurate, and again draws attention to the importance of real-time data on diagnostic testing intensity. However, even robust interventions like audit and feedback, if done in isolation, are unlikely to solve all of the challenges with test utilization. Multifaceted and coordinated interventions are likely helpful (18); for example by combining audit and feedback with test ordering decision support tools and de-adoption of obsolete or low-utility tests (8).

It is also important to discuss physician motivations for ordering tests. Survey respondents consistently cited clinical utility (94% overall) as an important consideration which is appropriate since a clinical question should prompt test ordering. Unlike clinical utility, only 24% of respondents cited cost as an important and 48% cited patient comfort; these findings are particularly surprising considering social desirability bias that may have inflated these numbers relative to true beliefs and practice (21). Our finding that many respondents do not consider cost may explain why interventions like displaying test prices to ordering providers have had modest impact (24). The lack of consideration of patient comfort is also worrisome in an era where patient-centered care and patient reported outcomes are increasingly recognized as important (25).

Finally, comparison across respondent groups (high vs low test utilizers and trainees vs attending physicians) warrants comment. Self-reported high utilizers of diagnostic investigations were even less likely than low/average utilizers to report considering cost (5% vs 27%, *p* = 0.04). This makes us wonder if education about cost is needed, or alternatively, if focusing on cost can ever be effective among providers who do not view fiscal considerations as important. Our finding that trainees were less likely than attendings to consider cost (42% vs 17%) and patient comfort (70% vs 41%) suggests that experience may also play a role in prioritizing these factors. Considering level of training may be important in designing future interventions to curtail over investigation.

Our study has several limitations. First, our study was conducted amongst Internal Medicine trainees and staff physicians at three Toronto teaching hospitals. While our results are likely to be generalizable to Internal Medicine trainees and staff at other Canadian teaching hospitals, extrapolating our findings to other clinical services (e.g., surgery, family medicine), other countries, or community hospitals may be premature. Rather we would suggest our findings need replication in other settings. Second, we relied on physician self-report of test ordering volumes and it is unclear how well self-reported testing behaviors correlate with actual practice. However, most physicians do not regularly receive data on their diagnostic testing utilization so self-perceptions are crucial. Finally, our study focused on inpatient internal medicine wards and it will be important to verify our results in other practice settings.

## Conclusions

The absence of real-time data on diagnostic testing utilization forces clinicians to rely upon self-perceptions. In our study, clinicians seem to recognize that over investigation is a problem, but few individuals self-identify as high test utilizers. We also found that a significant percentage of clinicians do not consider cost or patient discomfort when ordering tests. Our findings highlight challenges in reducing over-testing in the current era.

## Supplementary information

**Additional file 1: Table S1.** Survey Tool: Complete survey tool used during data collection

**Additional file 2: Table S2.** Question Specific Response Rates: Response rates for each individual question stratified by level of training (Attendings, Residents, Medical students)

**Additional file 3: Table S3.** Subgroup Analysis: Includes **Table S1.** (Residents compared to Medical Students), **Table S2.** (Senior Attendings compared to Junior Attendings), **Table S3.** (Male compared to Female participants), **Table S4.** (Self-identified High Utilizers of diagnostic tests with Likert 4–5 compared to Low Utilizers with Likert 1–2)

## Abbreviations

CT: (Computed Tomography)
ECG: (Electrocardiogram)
GIM: (General Internal Medicine)
MRI: (Magnetic Resonance Imaging)
