# Supplementary material for: Self-reported test ordering practices among Canadian internal medicine physicians and trainees: a multicenter cross-sectional survey
Source: BMC Health Serv Res. 2019 Nov 8;19:820. doi: 10.1186/s12913-019-4639-3 (PMC6842191; doi:10.1186/s12913-019-4639-3)
Supplement: Supplementary file 1 — Additional file 1: Table S1. Survey Tool: Complete survey tool used during data collection [file 12913_2019_4639_MOESM1_ESM.docx]

**Supplement 1 – Survey Tool**

**Definition of Terms:**

During the survey you will be asked to estimate the number of lab tests and other investigations you are ordering on your patients. What constitutes a single investigation will vary based on individual interpretation. For guidance, please refer to the following definitions and Table 1 and Table 2 below. When in doubt, use your best judgement. Always assume you are working on General Internal Medicine.

- **Laboratory Tests:** Any medical order which involves obtaining a sample from the patient (blood tests, cultures of bodily fluids) which is subsequently processed in a lab.
- **Other Investigations:** Any medical order which utilizes plain x-ray, CT, ultrasound, MRI or electrography or other means to provide information that is meant to guide diagnosis/management of the patient.
- **Clinically Significant Results:** Results of tests or investigations which would be considered important to the patient, and/or impact the clinical course or treatment plan for a patient.

**Table S1 Examples of a Single Laboratory Test**

| CBC |
| --- |
| Peripheral Blood Smear |
| Electrolytes (Na, K, Cl, HCO_3_) |
| Extended Electrolytes (Ca, Mg, PO_4_, Albumin) |
| Liver Studies (ALT, ALP, Bilirubin) |
| Coagulation Studies (INR/PTT/PT) |
| Serum Lactate |
| Cardiac Troponin |
| Blood Cultures x2 |
| Lumbar Puncture |
| Diagnostic Paracentesis |

**Table 2: Examples of a Single Other Investigations**

| Chest X-ray PA and Lateral |
| --- |
| ECG |
| EEG |
| Portable AP Chest Xray |
| Abdomen Xray Panel (eg. 3 views) |
| CT Chest/Abdomen/Pelvis |
| MRI Brain and Spine |
| CT Brain with or without angiogram |
| Bilateral Doppler Ultrasound of legs |
| Abdominal Ultrasound (including TVUS in women) |
| Echocardiogram |

**Part I: General Information**

1. Level of training (Check and complete appropriate section):

| □ Medical Student: | Level of Training (Circle): | CC3. CC4. |
| --- | --- | --- |
| □ Resident: | Level of Training (Circle): | PGY1, PGY2, PGY3, PGY4, PGY5, PGY6 |
|  |  |  |
|  | Residency Program (Circle): | Internal Medicine or Other: _____________ |
| □ Other: | Please Describe: | ____________________________________ |

2. Demographic Information:

| Age (in yrs): | ___________ |  |
| --- | --- | --- |
| Sex (circle): | Male. Female. Other. |  |

**Part II: Diagnostic Testing Practices**

1. How would you rate your diagnostic testing intensity (the number of tests you order on your patients), relative to your colleagues, on a scale of 1 to 5 with 3 being the average ordering intensity for a person **at your level of training**?

| 1 | 2 | 3 | 4 | 5 |  |  |
| --- | --- | --- | --- | --- | --- | --- |
| (much lower) | | | (average) |  | (much higher) | |

1. Do you feel that General Internal Medicine physicians order too many, too few, or just the right number of tests on their admitted inpatient services?

| 1 | 2 | 3 | 4 | 5 |  |  |
| --- | --- | --- | --- | --- | --- | --- |
| (too few tests) | | | (just right) |  | (too many tests) | |

1. Assuming you are the sole ordering physician, what is your best estimate of the average number of tests you order on a given patient during the first 24 hours of their hospital admission? (time zero is the time when the patient enters the Emergency Department).
   1. Number of Lab Tests (bloodwork): ______(Tests in 1^st^ 24 hours)
   2. Number of Other Investigations: ______(Tests in 1^st^ 24 hours)
2. Assuming you are the sole ordering physician, what is your best estimate of the number of **lab tests** you order on a daily basis (tests per patient per day) after the first 24 hours of admission?
   1. Number of Lab Tests (bloodwork): ______(Tests per day)
3. Assuming you are the sole ordering physician, what is your best estimate of the number of **other investigations** (examples in Table 2 on Page 2) you order over the course of a 7 day admission (total number)?
   1. Number of Other Investigations: ______(Total tests in a 7 day admission)
4. When estimating number of tests you are ordering in questions 3, 4 and 5 above, how confident are you in the accuracy of your estimates?

| 1 | 2 | | 3 | 4 | | 5 |  |  |
| --- | --- | --- | --- | --- | --- | --- | --- | --- |
| (not at all confident) | | | (somewhat confident) | | |  | (very confident) | |

1. How often do you consider the cost of a test when deciding what tests to order on your patients?

| 1 | 2 | | 3 | 4 | | 5 |  |  |
| --- | --- | --- | --- | --- | --- | --- | --- | --- |
| (never) | | | (50% of the time) | | |  | (always) | |

1. How often do you consider patient discomfort when deciding what tests to order on your patients?

| 1 | 2 | | 3 | 4 | | 5 |  |  |
| --- | --- | --- | --- | --- | --- | --- | --- | --- |
| (never) | | | (50% of the time) | | |  | (always) | |

1. How often do you consider clinical utility (i.e. whether or not a test will impact your management decisions) when deciding what tests to order?

| 1 | 2 | | 3 | 4 | | 5 |  |  |
| --- | --- | --- | --- | --- | --- | --- | --- | --- |
| (never) | | | (50% of the time) | | |  | (always) | |

1. What percentage of time in your work day do you spend deciding what tests to order?

| 0% | 10% | 20% | 30% | 40% | 50% | 60% | 70% | 80% | 90% | 100% |
| --- | --- | --- | --- | --- | --- | --- | --- | --- | --- | --- |
|  |  |  |  |  |  |  |  |  |  |  |

1. What is the most important thing you consider when ordering a test?

Please Describe: _________________________________________________________________________________________________________________________________________________________________________________________________________________________________________________
