# Supplementary material for: Self-reported test ordering practices among Canadian internal medicine physicians and trainees: a multicenter cross-sectional survey
Source: BMC Health Serv Res. 2019 Nov 8;19:820. doi: 10.1186/s12913-019-4639-3 (PMC6842191; doi:10.1186/s12913-019-4639-3)
Supplement: Supplementary file 2 — Additional file 2: Table S2. Question Specific Response Rates: Response rates for each individual question stratified by level of training (Attendings, Residents, Medical students) [file 12913_2019_4639_MOESM2_ESM.docx]

**Supplemental 2 – Question Specific Response Rates**

**Table 1 (Supplement 3):** Question specific response rates for attendings, residents, and medical students.

|  | **Attendings**  **(N=33)** | **Residents**  **(N=70)** | **Medical Students**  **(N=29)** |
| --- | --- | --- | --- |
| **Part I Q1:** Level Training | 33 (100%) | 70 (100%) | 29 (100%) |
| **Part I Q2:** Age | 31 (94%) | 70 (100%) | 29 (100%) |
| **Part I Q2:** Sex | 32 (97%) | 70 (100%) | 29 (100%) |
| **Part II Q1:** (Self-identified as a high utilizer of tests) | 33 (100%) | 69 (99%) | 28 (97%) |
| **Part II Q2:** Indicate that GIM providers order too many tests | 33 (100%) | 69 (99%) | 29 (100%) |
| **Part II Q3a:** Average number of lab tests per patient ordered in first 24 hours of admission | 33 (100%) | 69 (99%) | 28 (97%) |
| **Part II Q3b:** Average number of other tests ordered in first 24 hours | 32 (97%) | 69 (99%) | 26 (90%) |
| **Part II Q4:** Average number of lab tests per patient per day 1 through 7 of admission | 33 (100%) | 68 (97%) | 28 (97%) |
| **Part II Q5:** Average estimated number of other tests per day of admission | 33 (100%) | 68 (97%) | 27 (93%) |
| **Part II Q7:** Strongly considers cost when choosing lab tests | 33 (100%) | 70 (100%) | 29 (100%) |
| **Part II Q8:** Strongly considers patient comfort when choosing lab tests | 33 (100%) | 70 (100%) | 29 (100%) |
| **Part II Q9:** Strongly considers clinical utility when choosing lab tests | 33 (100%) | 69 (99%) | 28 (97%) |
| **Part II Q10:** Proportion of work day spent deciding what tests to order | 33 (100%) | 69 (99%) | 29 (100%) |
