# Supplementary material for: Self-reported test ordering practices among Canadian internal medicine physicians and trainees: a multicenter cross-sectional survey
Source: BMC Health Serv Res. 2019 Nov 8;19:820. doi: 10.1186/s12913-019-4639-3 (PMC6842191; doi:10.1186/s12913-019-4639-3)
Supplement: Supplementary file 3 — Additional file 3: Table S3. Subgroup Analysis: Includes Table S1. (Residents compared to Medical Students), Table S2. (Senior Attendings compared to Junior Attendings), Table S3. (Male compared to Female participants), Table S4. (Self-identified High Utilizers of diagnostic tests with Likert 4–5 compared to Low Utilizers with Likert 1–2) [file 12913_2019_4639_MOESM3_ESM.docx]

**Supplemental 3 – Subgroup Analysis**

**Table 1 (Supplement 3):** Responses from residents compared to medical students.

|  | **Residents (N = 70)^*^** | **Medical Students (N = 29)^*^** | **p** |
| --- | --- | --- | --- |
| **Median Age**, number (min-max) | 28  (24-37) | 25  (23-31) | - |
| **Female Sex**, number (%) | 30 (43%) | 13 (45%) | 0.86 |
| **Self-identified as a high utilizer of tests**^†^, number (%) | 9 (13%) | 4 (14%) | 1.0 |
| **Indicate that GIM providers order too many tests**^†^, number (%) | 55 (80%) | 17 (59%) | 0.03 |
| **Average number of lab tests per patient ordered in first 24 hours of admission**, number | 10.0 | 8.2 | 0.09 |
| **Average number of other**^‡^ **tests ordered in first 24 hours,** number | 2.7 | 2.7 | 0.92 |
| **Average number of lab tests per patient per day 1 through 7 of admission**, number | 4.2 | 5.1 | 0.52 |
| **Average estimated number of other**^‡^ **tests per day of admission,** number | 5.0 | 5.6 | 0.77 |
| **Strongly considers cost when choosing lab tests**^†^, number (%) | 10 (14%) | 7 (24%) | 0.24 |
| **Strongly considers patient comfort when choosing lab tests**^†^, number (%) | 31 (44%) | 10 (34%) | 0.37 |
| **Strongly considers clinical utility when choosing lab tests**^†^, number (%) | 64 (93%) | 25 (89%) | 0.67 |
| **Proportion of work day spent deciding what tests to order**, % | 29% | 37% | 0.03 |

^*^Percentages are based on question specific response rates rather than overall survey response rates.

^†^Response corresponds to 4 or 5 on a 5-point Likert Scale.

^‡^Structured definition for “other investigations” was provided including radiographic imaging, ECGs, etc.

**Table 2 (Supplement 3):** Responses from Senior (> 5 yr experience) compared to Junior (< 5 yr experience) attending physicians.

|  | **Senior Attendings  (N = 23)^*^** | **Junior Attendings (N = 9)^*^** | **p** |
| --- | --- | --- | --- |
| **Median Age**, number (min-max) | 45  (37-66) | 31  (28-38) | - |
| **Female Sex**, number (%) | 6 (27%) | 6 (67%) | 0.04 |
| **Self-identified as a high utilizer of tests**^†^, number (%) | 4 (17%) | 2 (22%) | 1.0 |
| **Indicate that GIM providers order too many tests**^†^, number (%) | 16 (70%) | 7 (78%) | 1.0 |
| **Average number of lab tests per patient ordered in first 24 hours of admission**, number | 14.5 | 7.0 | 0.01 |
| **Average number of other**^‡^ **tests ordered in first 24 hours,** number | 3.0 | 2.4 | 0.09 |
| **Average number of lab tests per patient per day 1 through 7 of admission**, number | 5.2 | 3.3 | 0.02 |
| **Average estimated number of other**^‡^ **tests per day of admission,** number | 3.5 | 8.4 | 0.22 |
| **Strongly considers cost when choosing lab tests**^†^, number (%) | 10 (43%) | 4 (44%) | 1.00 |
| **Strongly considers patient comfort when choosing lab tests**^†^, number (%) | 15 (65%) | 8 (89%) | 0.38 |
| **Strongly considers clinical utility when choosing lab tests**^†^, number (%) | 23 (100%) | 9 (100%) | 1.0 |
| **Proportion of work day spent deciding what tests to order**, % | 18% | 22% | 0.27 |

^*^Percentages are based on question specific response rates rather than overall survey response rates.

^†^Response corresponds to 4 or 5 on a 5-point Likert Scale.

^‡^Structured definition for “other investigations” was provided including radiographic imaging, ECGs, etc.

**Table 3 (Supplement 3):** Responses from male compared to female participants.

|  | **Male (N = 76)^*^** | **Female**  **(N = 53)^*^** | **p** |
| --- | --- | --- | --- |
| **Median Age**, number (min-max) | 28  (23-50) | 28  (23-66) | - |
| **Self-identified as a high utilizer of tests**^†^, number (%) | 8 (11%) | 11 (20%) | 0.15 |
| **Indicate that GIM providers order too many tests**^†^, number (%) | 56 (75%) | 40 (73%) | 0.80 |
| **Average number of lab tests per patient ordered in first 24 hours of admission**, number | 9.9 | 10.9 | 0.3 |
| **Average number of other**^‡^ **tests ordered in first 24 hours,** number | 2.8 | 2.8 | 0.97 |
| **Average number of lab tests per patient per day 1 through 7 of admission**, number | 4.8 | 4.2 | 0.38 |
| **Average estimated number of other**^‡^ **tests per day of admission,** number | 3.8 | 7.0 | 0.04 |
| **Strongly considers cost when choosing lab tests**^†^, number (%) | 17 (22%) | 14 (25%) | 0.68 |
| **Strongly considers patient comfort when choosing lab tests**^†^, number (%) | 37 (49%) | 26 (47%) | 0.87 |
| **Strongly considers clinical utility when choosing lab tests**^†^, number (%) | 75 (99%) | 46 (87%) | 0.01 |
| **Proportion of work day spent deciding what tests to order**, % | 27% | 29% | 0.23 |

^*^Percentages are based on question specific response rates rather than overall survey response rates.

^†^Response corresponds to 4 or 5 on a 5-point Likert Scale.

^‡^Structured definition for “other investigations” was provided including radiographic imaging, ECGs, etc.

**Table 4 (Supplement 3):** Self-identified high utilizers (Likert 4-5) compared to low utilizers (Liker 1-2).

|  | **High Utilizers^*^**  **of Tests (N = 19)** | **Low Utilizers^*^ of Tests**  **(N = 26)** | **p** |
| --- | --- | --- | --- |
| **Median Age**, number (min-max) | 29  (25-48) | 28  (23-66) | 0.73 |
| **Female Sex**, number (%) | 11 (58%) | 5 (19%) | 0.007 |
| **Indicate that GIM providers order too many tests**^†^, number (%) | 14 (74%) | 18 (69%) | 0.31 |
| **Average number of lab tests per patient ordered in first 24 hours of admission**, number | 12.42 | 8.42 | 0.06 |
| **Average number of other**^‡^ **tests ordered in first 24 hours,** number | 2.78 | 2.50 | 0.36 |
| **Average number of lab tests per patient per day 1 through 7 of admission**, number | 4.44 | 3.92 | 0.55 |
| **Average estimated number of other**^‡^ **tests per day of admission,** number | 1.02 | 2.81 | 0.07 |
| **Strongly considers cost when choosing lab tests**^†^, number (%) | 1 (5%) | 11 (42%) | 0.01 |
| **Strongly considers patient comfort when choosing lab tests**^†^, number (%) | 9 (47%) | 13 (50%) | 0.86 |
| **Strongly considers clinical utility when choosing lab tests**^†^, number (%) | 16 (84%) | 25 (96%) | 0.56 |
| **Proportion of work day spent deciding what tests to order**, % | 32% | 23% | 0.05 |

^*^Percentages are based on question specific response rates rather than overall survey response rates.

^†^Response corresponds to 4 or 5 on a 5-point Likert Scale.

^‡^Structured definition for “other investigations” was provided including radiographic imaging, ECGs, etc.
